# US Trends in COVID-19–Associated Hospitalization and Mortality Rates Before and After Reopening Economies

**DOI:** 10.1001/jamahealthforum.2021.1262

**Published:** 2021-06-25

**Authors:** Sumedha Gupta, Archelle Georgiou, Soumya Sen, Kosali Simon, Pinar Karaca-Mandic

**Affiliations:** 1 Department of Economics, Indiana University Purdue University, Indianapolis; 2 Carlson School of Management, Minneapolis, Minnesota; 3 Information & Decision Sciences, Carlson School of Management, Minneapolis, Minnesota; 4 O’Neill School of Public and Environmental Affairs, Indiana University, Bloomington; 5 Department of Finance, Carlson School of Management, Minneapolis, Minnesota

## Abstract

**Importance:**

After abrupt closures of businesses and public gatherings in the US in late spring 2020 due to the COVID-19 pandemic, by mid-May 2020, most states reopened their economies. Owing in part to a lack of earlier data, there was little evidence on whether state reopening policies influenced important pandemic outcomes—COVID-19–related hospitalizations and mortality—to guide future decision-making in the remainder of this and future pandemics.

**Objective:**

To investigate changes in COVID-19–related hospitalizations and mortality trends after reopening of US state economies.

**Design, Setting, and Participants:**

Using an interrupted time series approach, this cross-sectional study examined trends in per-capita COVID-19–related hospitalizations and deaths before and after state reopenings between April 16 and July 31, 2020. Daily state-level data from the University of Minnesota COVID-19 Hospitalization Tracking Project on COVID-19–related hospitalizations and deaths across 47 states were used in the analysis.

**Exposures:**

Dates that states reopened their economies.

**Main Outcomes and Measures:**

State-day observations of COVID-19–related hospitalizations and COVID-19–related new deaths per 100 000 people.

**Results:**

The study included 3686 state-day observations of hospitalizations and 3945 state-day observations of deaths. On the day of reopening, the mean number of hospitalizations per 100 000 people was 17.69 (95% CI, 12.54-22.84) and the mean number of daily new deaths per 100 000 people was 0.395 (95% CI, 0.255-0.536). Both outcomes displayed flat trends before reopening, but they started trending upward thereafter. Relative to the hospitalizations trend in the period before state reopenings, the postperiod trend was higher by 1.607 per 100 000 people (95% CI, 0.203-3.011; *P* = .03). This estimate implied that nationwide reopenings were associated with 5319 additional people hospitalized for COVID-19 each day. The trend in new deaths after reopening was also positive (0.0376 per 100 000 people; 95% CI, 0.0038-0.0715; *P* = .03), but the change in mortality trend was not significant (0.0443; 95% CI, −0.0048 to 0.0933; *P* = .08).

**Conclusions and Relevance:**

In this cross-sectional study conducted over a 3.5-month period across 47 US states, data on the association of hospitalizations and mortality with state reopening policies may provide input to state projections of the pandemic as policy makers continue to balance public health protections with sustaining economic activity.

## Key Points

**Question:**

Did the trends in COVID-19–related hospitalizations and deaths change after states reopened their economies?

**Findings:**

In this cross-sectional study of COVID-19–related hospitalizations and deaths across 47 US states between April 16 and July 31, 2020, the daily trend of hospitalizations after state reopenings was higher by 1.607 per 100 000 population. The change in the mortality rate trend was not significant.

**Meaning:**

These findings suggest that data on COVID-19–related hospitalizations and mortality trends can be used to guide health policy as states make decisions to open or close activities in response to this and future pandemics.

## Teaser

This cross-sectional study evaluates trends in hospitalization and death rates related to COVID-19 in US states between closures and reopenings of businesses and public gatherings in 2020.

## Introduction

In response to the COVID-19 pandemic, between March and April 2020, US states implemented nonessential business closures and stay-at-home (SAH) orders.^1^ These immediate policy responses were designed to mitigate transmission of SARS-CoV-2 that could otherwise exhaust hospital and intensive care unit capacity and thereby increase COVID-19–related mortality. These actions, together with voluntary social distancing, appear to have reduced the rates of new COVID-19 cases, deaths,^2^ and hospitalizations^3^ but were also associated with substantial increases in unemployment and other economic hardships.^4,5,6^

To alleviate financial harms, several states started reopening at the end of April 2020. By the middle of May 2020, most nonessential businesses had resumed at least some activities nationwide. A recent study reported an increase in human mobility following state reopenings.^7^ However, the effect of these policies on COVID-19–related hospital use and deaths remains unknown, partly owing to a lack of consistent data sources covering hospitalization data from the early pandemic stages onward.

We used daily data collected by the University of Minnesota COVID-19 Hospitalization Tracking Project^8^ since the early days of the pandemic on COVID-19–specific hospitalizations from US states together with daily COVID-19 state-level deaths data tracked by *The New York Times*.^9^ In a cross-sectional study using an interrupted time series design, we estimated changes in COVID-19–related hospitalizations and deaths before and after state reopenings. The 2 outcome variables were COVID-19–related hospitalizations and new deaths in state-day observations. This technique compared the trends in the outcome variable by day in the prereopening and postreopening periods.

## Methods

### Data Sources

Data on COVID-19–related hospitalizations were obtained from the University of Minnesota COVID-19 Hospitalization Tracking Project.^8^ These data were collected on a daily basis from states’ publicly available Department of Health websites and governor reports and have been used in other studies.^3,10,11,12,13^ The new COVID-19–related deaths per state per day were obtained from *The New York Times*^9^ and based on reports from state and local health agencies. Dates of state reopenings were obtained from Raifman et al^14^ and Nguyen and Simon.^15^ Data on average daily precipitation and temperature for seasonality controls were obtained from the US Environmental Protection Agency, Western Ecology Division laboratory website^16^ because these variables were shown to be associated with mobility and virus spread.^17,18^ The study was determined to be not human participants research and the need for informed consent was waived by the University of Minnesota institutional review board. This report followed the Strengthening the Reporting of Observational Studies in Epidemiology (STROBE) reporting guideline for cross-sectional studies.

### Study Measures and Population

We examined 2 COVID-19–specific outcome variables: current hospitalizations per capita and new COVID-19–related deaths per capita for each state-day. We sought to evaluate how trends in these outcomes varied before and after state reopenings.

We collected data on reopenings from *The New York Times*^19^ and verified through internet searches as well as details in Raifman et al 2020.^14^ A state’s initial reopening date was recorded as the date when the state governor’s office declared a state reopened (Table 1).

**Table 1.** State Reopening Dates in 2020^a^

State	Stay-at-home orders	Initial reopening	Expiration of stay-at-home orders	Public mask mandate
Alabama	April 4	April 30	April 31	July 16
Alaska^b^	March 28	April 24	NA	NA
Arizona	March 31	May 8	May 16	NA
Arkansas	NA	May 6	NA	July 20
California	March 19	May 8	NA	June 18
Colorado	March 26	May 1	August 22	July 16
Connecticut	March 23	May 20	May 21	April 20
Delaware	March 24	May 20	June 7	April 28
District of Columbia^b^	April 1	May 29	May 16	April 17
Florida	April 3	May 4	May 4	NA
Georgia	April 3	April 24	May 14	NA
Hawaii^b^	March 25	May 7	July 1	April 16
Idaho	March 25	May 1	May 1	NA
Illinois	March 21	May 1	May 30	May 1
Indiana	March 25	May 4	August 27	July 27
Iowa	NA	May 1	NA	NA
Kansas^c^	March 30	May 4	May 3	July 3
Kentucky	March 26	May 11	NA	May 11
Louisiana	March 23	May 15	June 5	July 13
Maine	April 1	May 1	NA	May 1
Maryland	March 30	May 15	NA	April 18
Massachusetts	March 24	May 18	NA	May 6
Michigan	March 24	April 24	June 13	April 27
Minnesota	March 28	April 27	May 18	July 24
Mississippi	April 3	April 27	June 1	NA
Missouri	April 6	May 4	May 4	NA
Montana	March 28	April 26	NA	NA
Nebraska	NA	May 4	NA	NA
Nevada	March 31	May 9	August 1	June 26
New Hampshire	March 28	May 11	September 1	NA
New Jersey	March 21	June 9	NA	April 8
New Mexico	March 24	May 16	August 29	May 15
New York	March 22	May 15	June 28	April 17
North Carolina	March 30	May 8	May 22	June 26
North Dakota	NA	May 1	NA	NA
Ohio	March 24	May 1	May 30	July 23
Oklahoma	NA	April 2	NA	NA
Oregon	March 23	May 15	NA	July 1
Pennsylvania	April 1	May 8	June 5	July 1
Rhode Island	March 28	May 9	May 23	April 18
South Carolina	April 7	April 20	August 10	NA
South Dakota	NA	May 1	NA	NA
Tennessee	April 1	April 27	August 30	NA
Texas	April 2	May 16	May 16	July 3
Utah	March 27	May 1	May 2	April 10
Vermont	March 24	April 27	August 16	August 1
Virginia	March 30	May 15	June 10	May 29
Washington	March 23	May 5	July 2	June 26
West Virginia	March 24	May 4	NA	July 7
Wisconsin	March 25	April 29	May 26	August 1
Wyoming	NA	May 1	NA	NA

Abbreviation: NA, not available.

^a^ Dates of state reopenings, stay-at-home orders and mask mandates were obtained from Raifman et al^14^ and Nguyen and Simon.^15^ Data on COVID-19–specific hospitalizations were obtained from the University of Minnesota COVID-19 Hospitalization Tracking Project.^8^ The new COVID-19 deaths per state per day were obtained from *The New York Times*.^9,19^

^b^ Data on hospitalizations and deaths not available for analysis.

^c^ Data on hospitalizations not available for analysis.

Our study population included 3686 state-day observations from 47 US states that reported data on hospitalizations between April 16 and July 31, 2020.

### Statistical Analysis

To investigate the association of reopening with the outcome variables, we estimated an interrupted time series specification that captured both the changes in levels and the trends for the outcome variables (eMethods in the Supplement provides detailed regression specification). These specifications adjusted for systematic differences between states using state indicators and included calendar date indicators to account for changes that were constant across states but varied over time. The key exposure variables were a daily linear time trend, an indicator for the days after the reopening date (ie, postreopening), and an interaction of the time trend and this postreopening indicator. A positive, statistically significant coefficient estimate on this interaction indicated that the trend in the outcome variables increased after reopenings. Because the median incubation period of novel SARS-Cov-2 is 5 days and the median time between symptom onset to hospitalization is 7 days,^3^ we excluded these 12 days from our analysis (a washout period) after the reopening day. Our analysis also adjusted for average daily precipitation and average daily temperature in each state to control for any seasonality in human mobility that may have affected COVID-19 transmission. Heteroscedasticity robust SEs were clustered at the state level.

We conducted several sensitivity analyses to confirm the robustness of our estimates.^20^ First, we tested alternative washout periods of 8 days (from reopening), corresponding to the 25th percentile of incubation, and 15 days (from reopening), corresponding to the 75th percentile of the incubation period (from infection to hospitalization).^21,22^ Second, to examine whether we could estimate state reopening associations similar in magnitude to ours by chance, we randomized the timing of state reopenings to alternative pseudo start dates in the preintervention time continuum. We expected that state reopenings take effect only after the actual reopening date, with no significant effect earlier. We randomized the date of state reopenings 1000 times. The *P* values from the randomization inference exercises were the fraction of estimated coefficients that were as large as those estimated for the true state reopening dates.^23^ Third, to account for counties with different reopening policies than the state, an alternative specification defined state reopenings as the share of state population living in counties that opened on state reopening dates (population living in counties that reopened/state population) for each state-calendar date observation using county level policies from the Centers for Disease Control and Prevention.^24^

All analyses were performed with Stata, version 16.1 (StataCorp LLC). The 95% CIs around estimates reflect 0.025 in each tail or *P* ≤ .05 and *P* values are from 2-tailed *t* tests of the coefficients from regression models.

## Results

**Figure 1.**
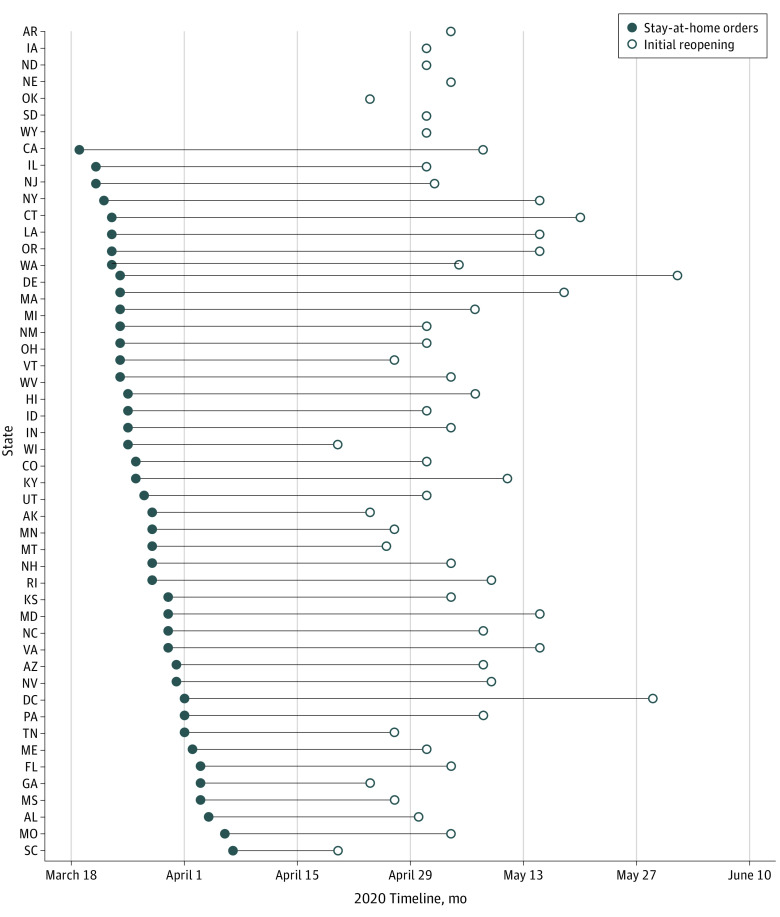
Timeline of State Reopenings Seven states (AR, IA, NE, ND, OK, SD, and WY) did not implement official stay-at-home orders during the study period, although at least some of these states issued orders for nonessential business closures and other guidance to induce social distancing. All states, including the 7 that did not implement stay-at-home orders, had official state reopenings.

Figure 1 displays each state’s reopening date. Between April 20 (South Carolina, Wisconsin) and June 1, 2020 (Delaware), all states reopened. Details of state reopening dates and study samples are presented in Table 1.

Unadjusted daily rates of current hospitalization and new deaths varied extensively across states both before and after reopenings. Before state reopenings, the mean hospitalization rate per 100 000 people was 25.52 (interquartile range [IQR], 7.00-37.62), and the corresponding rate after reopenings was 13.08 (IQR, 5.41-16.09). The mean new COVID-19 death rate before reopenings was 0.63 (IQR, 0.10-0.87), and the corresponding rate after reopenings was 0.22 (IQR, 0.03-0.28).

**Figure 2.**
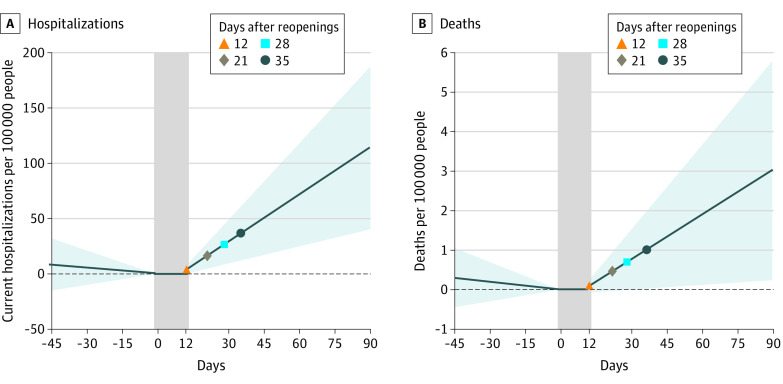
Interrupted Time Series Estimates of Adjusted Change in Rates of COVID-19–Related Hospitalizations and Deaths Associated With State Initial Reopenings Changes in hospitalizations (A) and deaths (B) relative to the day of initial reopening. The vertical gray bars capture day 0 (day of reopening) through day 12 (end of washout period). The shaded areas represent 95% CIs.

Interrupted time series estimates are presented in Table 2, which displays the trend and the change in trend of the outcome variables associated with state reopening. Before the state reopenings, the trend in daily hospitalization rate per 100 000 people was not statistically significantly different from 0 (−0.191; 95% CI, −0.720 to 0.339; *P* = .47). After the reopenings (incorporating the 12-day washout period corresponding to the median effective date),^3^ the hospitalization trend increased to 1.417 (95% CI, 0.515-2.318; *P* = .003), which resulted in a statistically significant increase of 1.607 (95% CI, 0.203-3.011; *P* = .03) in daily time trend of hospitalization rate associated with the state reopening. The mean hospitalization rate on the day of reopening was 17.69 per 100 000 people. By 12 days after reopening, the hospitalization rate increased by 3.96 (95% CI, −0.23 to 8.14), although the increase was not statistically significant. The estimated increase in corresponding rates was 16.70 (95% CI, 4.74-28.66) after 21 days and 26.62 (95% CI, 8.41-44.83) after 28 days of reopening (Figure 2). Overall, the estimated change of 1.607 additional hospitalizations per 100 000 people associated with the reopenings suggested that nationwide reopenings were associated with 5319 additional people hospitalized for COVID-19 in a given day (1.607 multiplied by the US population of 331 002 651, divided by 100 000) (Table 2 and Figure 2).

**Table 2.** Adjusted Change in Trends: Rates of COVID-19 Hospitalizations and Deaths^a^

Variable	Estimate (95% CI)	*P* value
Hospitalizations (n = 3686)		
Prereopening daily trend	−0.191 (−0.720 to 0.339)	.47
Postreopening daily trend	1.417 (0.515 to 2.318)	.003
Change in trend	1.607 (0.203 to 3.011)	.03
Unadjusted mean on day of reopening	17.69 (12.54 to 22.84)	NA
Deaths (n = 3945)		
Prereopening daily trend	−0.0067 (−0.0233 to 0.0100)	.43
Postreopening daily trend	0.0376 (0.0038 to 0.0715)	.03
Change in trend	0.0443 (−0.0048 to 0.0933)	.08
Unadjusted mean on day of reopening	0.395 (0.255 to 0.536)	NA

Abbreviation: NA, not applicable.

^a^ Adjusted estimate from the interrupted time series analysis of the association between state reopenings and rates of COVID-19 hospitalizations and deaths relative to the day of reopening. Sample included daily data from 47 US states for COVID-19–related hospitalizations per 100 000 people (some dates missing, detailed in Table 1) and COVID-19–related daily new deaths per 100 000 people, April 16, 2020, to July 31, 2020. Regressions included controls for daily average temperature and precipitation and indicators for state and calendar date. Heteroscedasticity robust SEs were clustered at the state level.

The trend in the new daily death rate per 100 000 people was not significantly different from 0 before reopening (−0.0067; 95% CI, −0.0233 to 0.0100; *P* = .43). Although the trend was positive and statistically significant after reopenings (0.0376; 95% CI, 0.0038-0.0715; *P* = .03), the difference from before to after reopening was not statistically significant (0.0443; 95% CI, −0.0048 to 0.0933; *P* = .08) (Table 2). The mean daily new deaths per 100 000 people on the day of reopening was 0.395 (95% CI, 0.255-0.536). Although the death rate started increasing after reopening, increasing by 0.10 (95% CI, −0.08 to 0.28) 12 days after reopening, the increase became statistically significant only after 35 days. The death rate increased by 0.96 (95% CI, 0.03-1.89) 35 days after reopening (Figure 2).

States varied in the nature of reopening,^14^ and in additional analyses, we distinguished between states that immediately reopened all economic sectors (outdoor recreation, retail, restaurant, worship, personal care, entertainment, and industry activities) vs those that used a phased approach to reopenings.^15^ Sixteen states implemented immediate reopenings (n = 1260) of all businesses; 31 states implemented phased reopenings (n = 2426). In 37 states, SAH orders were still in effect at the time of state reopenings (n = 2939); in 10 states, SAH orders had expired on or before the state reopenings (n = 747). In 35 states, public mask mandates were not in effect at the time of state reopenings (n = 2714); 12 states had adopted public mask mandates before or in conjunction with reopenings (n = 972). We found that states with phased reopenings had both higher rates of hospitalization on the day of reopening relative to those with immediate reopenings (20.93; 95% CI, 13.92-9.95 vs 9.95; 95% CI, 7.01-12.90) and a higher change in hospitalization trend after reopening (1.403; 95% CI, −0.033 to 2.840; *P* = .06 vs −0.659; 95% CI, −2.176 to 0.859 per day, a difference of 2.062; 95% CI, 0.469-3.655; *P* = .01) (Table 3). States with an SAH order at the time of reopening also had higher hospitalization rates relative to those with an expired order, although the CIs were large (18.93; 95% CI, 12.95-24.90 vs 13.47; 95% CI, 1.67-25.28), and the relative increase in hospitalizations associated with reopening was also higher in states with an SAH order at the time of reopening relative to those with an expired SAH order (1.492; 95% CI, 0.0534-2.931; *P* = .04 vs −0.011; 95% CI, −1.103 to 1.080 per day, a difference of 1.504; 95% CI, 0.432-2.576; *P* = .01). We did not find significant differences in the change in hospitalization trend associated with reopening between states with and without a mask mandate at the time of reopening.

**Table 3.** Adjusted Change in Trends of Hospitalization Rate by Reopening Characteristics^a^

Observed time trend	Reopening characteristic																	
	Phased reopenings		Immediate reopenings		Difference		SAH orders in place		Expired SAH		Difference		Mask mandate		No mask mandate		Difference	
	Estimate (95% CI)	*P* value	Estimate (95% CI)	*P* value	Estimate (95% CI)	*P* value	Estimate (95% CI)	*P* value	Estimate (95% CI)	*P* value	Estimate (95% CI)	*P* value	Estimate (95% CI)	*P* value	Estimate (95% CI)	*P* value	Estimate (95% CI)	*P* value
Prereopening daily trend	−0.206 (−0.731 to 0.32)	.44	0.875 (0.206 to 1.545)	.01	−1.081 (−1.91 to −0.253)	.01	−0.157 (−0.694 to 0.380)	.56	0.608 (0.134 to 1.081)	.01	−0.765 (−1.30 to −0.23)	.01	−0.442 (−1.114 to 0.23)	.19	0.170 (−0.41 to 0.749)	.56	−0.612 (−1.56 to 0.335)	.20
Postreopening daily trend	1.198 (0.240 to 2.155)	.02	0.217 (−0.69 to 1.124)	.63	0.981 (0.206 to 1.755)	.01	1.335 (0.406 to 2.265)	.01	0.596 (−0.10 to 1.294)	.09	0.739 (0.193 to 1.285)	.01	0.644 (−0.526 to 1.82)	.27	0.443 (−0.335 to 1.24)	.27	0.201 (−0.821 to 1.223)	.69
Change in trend	1.403 (−0.033 to 2.84)	.06	−0.659 (−2.18 to 0.859)	.39	2.062 (0.469 to 3.655)	.01	1.492 (0.0534 to 2.931)	.04	−0.011 (−1.103 to 1.08)	.98	1.504 (0.432 to 2.576)	.01	1.086 (−0.696 to 2.87)	.23	0.273 (−1.012 to 1.56)	.67	0.813 (−1.45 to 2.771)	.41
Unadjusted mean on day of reopening	20.93 (13.92 to 27.94)	NA	9.95 (7.01 to 12.90)	NA	10.97 (0.06 to 21.88)	NA	18.93 (12.95 to 24.90)	NA	13.47 (1.67 to 25.28)	NA	5.45 (−6.87 to 17.78)	NA	33.557 (20.30 to 46.80)	NA	11.03815 (8.09 to 13.97)	NA	22.52 (13.5 to 31.55)	NA

Abbreviations: NA, not applicable; SAH, stay at home.

^a^ The analytic study sample included 3686 state-day observations from 47 US states for COVID-19 related hospitalizations per 100 000 people (n = 3686). Sixteen states implemented immediate reopenings (n = 1260) of all businesses; 31 states implemented phased reopenings (n = 2426). In 37 states, SAH orders were still in effect at the time of state reopenings (n = 2939); in 10 states, SAH orders had expired on or before the state reopenings (n = 747). In 35 states, public mask mandates were not in effect at the time of state reopenings (n = 2714); 12 states had adopted public mask mandates before or in conjunction with reopenings (n = 972). Regressions on stratified samples included controls for daily average temperature and precipitation, and indicators for state and calendar date. Heteroscedasticity robust SEs were clustered at the state level.

Our results were robust to a battery of sensitivity tests. First, our results were qualitatively similar when using alternative 8-day and 15-day washout periods corresponding to the 25th and 75th percentile of incubation period (from infection to hospitalization) (eTable 1 in the Supplement). Second, when we randomized the timing of state reopenings to alternative pseudo start dates in the preintervention time continuum, the reported *P* values capturing the fraction of estimated coefficients that were as large as those estimated for the true state reopening dates were generally less than 5% of the cases (eTable 2 in the Supplement). This finding suggests that one is very unlikely to estimate an association with state reopenings similar in magnitude to those we estimated using true state reopening times purely by chance. Third, our results did not qualitatively change if state reopenings were captured as the share of state population living in counties that opened on state reopening dates for each state-calendar date observation; we continued to find statistically significant increases in trends in both hospitalizations and deaths following state reopenings with this alternative reopening policy specification (eTable 3 in the Supplement).

## Discussion

In this cross-sectional study, we addressed a gap in the literature to examine whether state policies implemented in spring 2020 to protect hospital capacity and minimize deaths due to COVID-19 were associated with hospitalizations and mortality. This gap in knowledge is a shortcoming because the pandemic requires continued reassessment of the optimal level of activities resumption. We found that, prior to reopening, there was a flat trend in current COVID-19–related hospitalizations and new daily deaths regardless of state reopening decisions that was not significantly different from 0; however, the hospitalization and mortality rates were positive after reopening. Earlier research showed that during the closure period (before reopening), reduced mobility was associated with reductions in hospitalizations^3^ and deaths.^25^ Our findings that hospitalization and mortality trends were positive after reopenings supports the findings from studies showing reopenings were substantially associated with higher mobility,^26,27,28,29^ emphasizing the health outcomes associated with reopenings.

When we stratified our analyses by state characteristics, we found that hospitalization rates increased more in states with an active SAH order in place at the time of reopening and in states with phased reopenings. Although our data were not able to offer a definitive explanation for these findings, our findings showed that states that reopened but maintained some interventions to mitigate the spread of COVID-19 (phased reopening, SAH order, and/or mask mandates) had higher levels of hospitalization rates before reopening (Table 3).

### Limitations

The study has limitations. The cross-sectional study design of our study provides associations and not causally interpretable estimates given the possible nonrandomized nature of state policy decision. There were other data limitations as well. First, states varied in their reporting of COVID-19–related hospitalizations, and some may have included suspected cases in their total. When both suspected and confirmed hospitalization data were available, we included only confirmed cases. Second, some hospitalizations may have included cases in which COVID-19 was a contributing, but not primary, diagnosis. In addition, although it would be informative to also examine other COVID-19–related hospitalization outcomes, such as intensive care unit and ventilator use, these data were available for a considerably smaller subset of states. Nevertheless, our data from the University of Minnesota COVID-19 Hospitalization Tracking Project offer a comprehensive examination of the outcome variables we studied from all states that reported them capturing the early stages of the pandemic, before and after the state reopenings. The Department of Health and Human Services started releasing data on hospital capacity (hospital beds and intensive care unit beds occupied by patients with COVID-19) at the state level starting late July 2020^30^ and at the hospital facility level starting December 2020.^31^ However, those data sets do not allow capturing the time frame early enough to study state reopenings in April and May.

## Conclusions

To our knowledge, this is the first study to quantify the association between COVID-19–related hospitalizations, deaths, and state reopenings in the US. Because a major risk of COVID-19 was exceeding the capacity of the health care infrastructure, a better understanding of the projections of COVID-19–related health care use is valuable, especially for the future waves of the pandemic. Our findings provide quantifiable evidence to hospital systems, health care professionals, and policy makers to help project and remain aware of needs for ensuring adequate hospital capacity and care as states continue to further open or close activities.

## Notes

Supplement. **eMethods.** Description of Multivariable Regression Approach **eTable 1.** Adjusted Change in Trends: Rates of COVID-19 Hospitalizations and Deaths With Alternative 8-Day and 15-Day Washout Periods Corresponding to 25th and 75th Percentile of Incubation Period (From Infection to Hospitalization) Respectively **eTable 2.** Randomization Inference of Adjusted Change in Trends: Rates of COVID-19 Hospitalizations and Deaths **eTable 3.** Adjusted Change in Trends: Rates of COVID-19 Hospitalizations and Deaths, With Reopening Defined as the Share of State Population Impacted by State Initial Reopenings
