# Supplementary material for: US Trends in COVID-19–Associated Hospitalization and Mortality Rates Before and After Reopening Economies
Source: JAMA Health Forum. 2021 Jun 25;2(6):e211262. doi: 10.1001/jamahealthforum.2021.1262 (PMC8796994; doi:10.1001/jamahealthforum.2021.1262)
Supplement: Supplement. — eMethods. Description of Multivariable Regression Approach eTable 1. Adjusted Change in Trends: Rates of COVID-19 Hospitalizations and Deaths With Alternative 8-Day and 15-Day Washout Periods Corresponding to 25th and 75th Percentile of Incubation Period (From Infection to Hospitalization) Respectively eTable 2. Randomization Inference of Adjusted Change in Trends: Rates of COVID-19 Hospitalizations and Deaths eTable 3. Adjusted Change in Trends: Rates of COVID-19 Hospitalizations and Deaths, With Reopening Defined as the Share of State Population Impacted by State Initial Reopenings [file jamahealthforum-e211262-s001.pdf]

Supplementary Online Content

Gupta S, Georgiou A, Sen S, Simon K, Karaca-Mandic P. US trends in COVID-19–associated hospitalization and mortality rates before and after reopening economies. *JAMA Health Forum*. 2021;2(6):e211262. doi:10.1001/jamahealthforum.2021.1262

eMethods. Description of Multivariable Regression Approach

eTable 1. Adjusted Change in Trends: Rates of COVID-19 Hospitalizations and Deaths With Alternative 8-Day and 15-Day Washout Periods Corresponding to 25th and 75th Percentile of Incubation Period (From Infection to Hospitalization) Respectively

eTable 2. Randomization Inference of Adjusted Change in Trends: Rates of COVID-19 Hospitalizations and Deaths

eTable 3. Adjusted Change in Trends: Rates of COVID-19 Hospitalizations and Deaths, With Reopening Defined as the Share of State Population Impacted by State Initial Reopenings

This supplementary material has been provided by the authors to give readers additional information about their work.

© 2021 Gupta S et al. *JAMA Health Forum.*

eMethods. Description of Multivariable Regression Approach

The objective of the analysis was to assess the impact of state re-openings on rates of COVID-19 hospitalizations and deaths. To do so, we used data at the state-calendar date level on COVID-19 hospitalizations and deaths.

Model of COVID-19 Hospitalizations and Deaths Throughout, we use $s = 1 \ldots S$ to index states and $t = 1 \ldots T$ to index calendar dates. We estimated a regression of the following form for COVID-19 hospitalizations and deaths:

$$Y_{st} = \beta_0 + \beta_1 T_t + \beta_2 X_t + \beta_3 Z_t + \gamma_s + \tau_t + \varepsilon_{st} \quad (1)$$

In this regression model, Y_{st} represents the dependent variables of interest. In models of hospitalizations, Y_{st} is the number of COVID-19 related hospitalizations per 100,000 persons in state s on calendar date t. In models of COVID-19 deaths, Y_{st} is the number of COVID-19 related deaths per 100,000 persons in state s on calendar date t. We include indicator variables (fixed effect controls) for state and for week. We also include controls for state average temperature and precipitation which are noted predictors of human mobility and thus cases leading to hospitalizations and deaths. Heteroscedasticity robust standard errors are clustered at the state level. On the right-hand side, β_0 is the intercept capturing the level of the dependent variable at the start of the study period, T_t is the time since start of the study period with β_1 capturing the pre-re-opening trend in the dependent variable, X_t is a dummy (indicator) variable representing the re-opening period (prior to re-opening $X_t = 0$, post re-opening $X_t = 1$) with β_2 denoting the change in the intercept of the outcomes immediately after re-openings, and Z_t is the time since re-opening and β_3 captures the post-re-opening trend in the dependent variable. Thus, difference in the estimated intercept coefficients $(\widehat{\beta_2} - \widehat{\beta_0})$ is the change in the level of the dependent variable following state re-openings and difference in the estimated slope coefficients $(\widehat{\beta_3} - \widehat{\beta_1})$ is the change in the trend of the dependent variable following state re-openings.

© 2021 Gupta S et al. *JAMA Health Forum.*

eTable 1. Adjusted change in trends: rates of COVID-19 hospitalizations and deaths with alternative 8-day and 15-day washout periods corresponding to 25th and 75th percentile of incubation period (from infection to hospitalization) respectively.

	(1)	(2)	(3)	(4)
	8-day washout period		15-day washout period	
		P-value		P-value
**Hospitalizations (N=3686)**				
Pre-Re-Opening Daily Trend	0.167	0.51	-0.302	0.27
	[-0.337, 0.671]		[-0.850, 0.246]	
Post-Re-Opening Daily Trend	1.371	0.003	1.442	0.003
	[0.492, 2.249]		[0.529, 2.356]	
Change in Trend	1.203	0.07	1.745	0.02
	[-0.099, 2.505]		[0.300, 3.189]	
Unadjusted mean on day of re-opening	17.69			
	[12.54, 22.84]			
**Deaths (N=3945)**				
Pre-Re-Opening Daily Trend	0.0031	0.72	-0.0086	0.33
	[-0.0134, 0.0197]		[-0.026, 0.009]	
Post-Re-Opening Daily Trend	0.0365	0.03	0.0386	0.03
	[0.0034, 0.0696]		[0.0044, 0.073]	
Change in Trend	0.0334	0.14	0.0471	0.07
	[-0.0119, 0.0786]		[-0.0036, 0.098]	
Unadjusted mean on day of re-opening	0.395			
	[0.255, 0.536]			

Note: Table presents the adjusted estimate from the interrupted time series analysis of the association between state-re-openings and rates of COVID-19 hospitalization and rates of deaths relative to the day of re-opening. The analytic study sample includes daily data from 47 U.S. states for COVID-19 related hospitalizations per 100,000 people (N=3686) and new COVID-19 related deaths per 100,000 (N=3945). The observation window was April 16, 2020 – July 31, 2020, but with gaps. Refer to Table 1 for details of states captured in the sample. Regression included controls for daily average temperature and precipitation, and indicators for state and calendar date. Heteroscedasticity robust standard errors were clustered at state level. Columns (1)-(2) presents sensitivity checks with 8-day (25th percentile of the incubation period from infection to hospitalization) and columns (3)-(4) test for sensitivity of results to a 15-day (75th percentile of the incubation period from infection to hospitalization) washout periods.

© 2021 Gupta S et al. *JAMA Health Forum.*

eTable 2: Randomization Inference of Adjusted change in trends: rates of COVID-19 hospitalizations and deaths

	(1)	(2)
	Estimated coefficients with actual date of state re-openings	Share of Estimates using Randomized State Re-opening Dates that are Larger Than Estimated Coefficients with actual state re-openings (P-value)
Hospitalization (N=3686), 1000 randomizations of state re-opening dates		
Pre-Re-Opening Daily Trend	-0.191	0.001
Post-Re-Opening Daily Trend	1.417	0.04
Change in Trend	1.607	0.02
Deaths (N=3945), 1000 randomizations of state re-opening dates		
Pre-Re-Opening Daily Trend	-0.0067	0.01
Post-Re-Opening Daily Trend	0.0376	0.05
Change in Trend	0.0443	0.03

Note: Table presents the adjusted estimate from the interrupted time series analysis of the association between state-re-openings and rates of COVID-19 hospitalization and rates of deaths relative to the day of re-opening. The analytic study sample includes daily data from 47 U.S. states for COVID-19 related hospitalizations per 100,000 people (N=3686) and COVID-19 related deaths per 100,000 (N=3945). The observation window was April 16, 2020 – July 31, 2020, but with gaps. Refer to Table 1 for details of states captured in the sample. Regression included controls for daily average temperature and precipitation, and indicators for state and calendar date. Heteroscedasticity robust standard errors were clustered at state level. Column (1) presents coefficient estimates using the actual dates of state re-openings (refer Table 2 for details). Column (2) presents p-values from randomization inference exercise where we randomized timing of treatment to alternative ‘pseudo’ start dates in the pre-intervention time continuum. We expected that state re-openings should take effect only after actual re-opening date, with no significant effect earlier. We randomized the date of state re-openings 1000 times. The p-values from the randomization inference exercises are the fraction of estimated coefficients that are as large as those estimated for the true state re-opening dates. The p-values show that, generally, change in trend from randomized re-opening dates are as large as our main estimates in less than 5 percent of the cases where re-opening timing is randomly assigned across states.

© 2021 Gupta S et al. *JAMA Health Forum.*

eTable 3: Adjusted change in trends: rates of COVID-19 hospitalizations and deaths, with reopening defined as the share of state population impacted by state initial reopenings.

	(1)	(2)
		P-value
Hospitalizations (N=3686)		
Pre-Re-Opening Daily Trend	-0.210	0.02
	[-0.0334, -0.386]	
Post-Re-Opening Daily Trend	0.308	0.09
	[-0.0443,0.661]	
Change in Trend	0.518	0.05
	[0.004,1.032]	
Unadjusted mean on day of re-opening	17.69	
	[12.54, 22.84]	
Deaths (N=3945)		
Pre-Re-Opening Daily Trend	-0.007	0.02
	[-0.0008,-0.121]	
Post-Re-Opening Daily Trend	0.0098	0.06
	[-0.0004,0.0199]	
Change in Trend	0.0162	0.04
	[0.0007,0.0318]	
Unadjusted mean on day of re-opening	0.395	
	[0.255, 0.536]	

Note: Table presents the adjusted estimate from the interrupted time series analysis of the association between state-re-openings and rates of COVID-19 hospitalization and deaths relative to the day of re-opening. The analytic study sample includes daily data from 47 U.S. states for COVID-19 related hospitalizations per 100,000 people (N=3686) and COVID-19 related deaths per 100,000 people (N=3945). The observation window was April 16, 2020 – July 31, 2020, but with gaps. Refer to Table 1 for details of states captured in the sample. Regression included controls for daily average temperature and precipitation, indicators for state and calendar date. Heteroscedasticity robust standard errors were clustered at state level. Instead of defining state re-opening as an indicator variable, state re-openings were defined as the share of state population living in counties that opened on state re-opening dates (population living in counties that re-opened/state population) for each state-calendar date cell using county level information of county level policies from the CDC (https://catalog.data.gov/dataset/u-s-state-and-territorial-stay-at-home-orders-march-15-september-14-by-county-by-day-c2aad)

© 2021 Gupta S et al. *JAMA Health Forum.*
